# Affinity Capillary Electrochromatography of Molecularly Imprinted Thin Layers Grafted onto Silica Capillaries Using a Surface-Bound Azo-Initiator and Living Polymerization

**DOI:** 10.3390/polym10020192

**Published:** 2018-02-15

**Authors:** Cristina Giovannoli, Cinzia Passini, Fabio Di Nardo, Laura Anfossi, Claudio Baggiani, Ian A. Nicholls

**Affiliations:** 1 Laboratory of Bioanalytical Chemistry, Department of Chemistry, Via Giuria 7, University of Torino, 10125, Torino, Italy; cinzia.passini@unito.it (C.P.); fabio.dinardo@unito.it (F.D.N.); laura.anfossi@unito.it (L.A.); claudio.baggiani@unito.it (C.B.); 2 Bioorganic & Biophysical Chemistry Laboratory, Centre for Biomaterials Chemistry, Department of Chemistry & Biomedical Sciences, Linnaeus University, SE-39182 Kalmar, Sweden; ian.nicholls@lnu.se

**Keywords:** molecularly imprinted polymers, capillary electrophoresis, controlled/living radical polymerization, 2,4,5-trichlorophenoxyacedic acid, warfarin

## Abstract

Molecularly imprinted thin layers were prepared in silica capillaries by using two different surface polymerization strategies, the first using 4,4′-azobis(4-cyanovaleric acid) as a surface-coupled radical initiator, and the second, S-carboxypropyl-S’-benzyltrithiocarbonate as a reversible addition-fragmentation chain transfer (RAFT) agent in combination with 2,2′-azobisisobutyronitrile as a free radical initiator. The ability to generate imprinted thin layers was tested on two different polymerization systems: (i) a 4-vinylpyridine/ethylene dimethacrylate (4VP-EDMA) in methanol-water solution with 2,4,5-trichlorophenoxyacetic acid (2,4,5-T) as a template; and (ii) methacrylic acid/ethylene dimethacrylate (MAA-EDMA) in a chloroform solution with warfarin as the template molecule. The binding properties of the imprinted capillaries were studied and compared with those of the corresponding non-imprinted polymer coated capillaries by injecting the template molecule and by measuring its migration times relative to a neutral and non-retained marker. The role of running buffer hydrophobicity on recognition was investigated by studying the influence of varying buffer acetonitrile concentration. The 2,4,5-T-imprinted capillary showed molecular recognition based on a reversed phase mechanism, with a decrease of the template recognition in the presence of higher acetonitrile content; whereas warfarin-imprinted capillaries showed a bell-shaped trend upon varying the acetonitrile percentage, illustrating different mechanisms underlying imprinted polymer-ligand recognition. Importantly, the results demonstrated the validity of affinity capillary electrochromatography (CEC) to screen the binding properties of imprinted layers.

## 1. Introduction

Molecular imprinting is a popular approach for the preparation of tailor-made polymer-based molecular recognition systems for a predetermined molecular target. As a consequence, molecularly-imprinted polymers (MIPs) have attracted significant attention in many application fields including solid-phase extraction, chemical sensing, drug delivery, and catalysis [1,2,3,4].

Nowadays, in MIP technology, the most popular synthetic approach is still based on bulk free radical polymerization because of its compatibility with a wide range of monomers and templates, as well as for the mild reaction conditions required. However, this approach is characterized by poor control of polymer morphology and by a high resistance to mass-transfer in the resulting polymers. In order to overcome these drawbacks, and to move towards more efficient formats with controlled morphology, with defined nano-scale architectures and improved kinetic and thermodynamic binding properties, more advanced synthetic strategies have become one of the main needs [5,6,7]. 

Among the most attractive synthesis techniques in nanomaterial design is the “grafting from” approach. Basically, it relies on the covalent attachment of a radical initiator monolayer onto a solid surface, followed by a radical initiated polymerization at the surface-solution interface. In such a way, the immobilized radical initiator allows the growth of a polymer layer, while no unwanted polymeric growth takes place in bulk solution [8,9]. The combination of the “grafting from” approach with controlled living radical polymerization methods has proven to be a versatile strategy to fine tune the growth of the polymer layer. Controlled living radical polymerization can be achieved by using metal-catalysed atom transfer radical polymerization (ATRP), dithiocarbamate-based polymerization (iniferter polymerization), or reversible addition-fragmentation chain transfer (RAFT) polymerization [10,11]. 

Controlled surface imprinting can be considered a useful approach not only to obtain novel nano-structured imprinted materials, but also to design new devices and separation tools at the micro- and nanometer levels [12,13]. Among these, molecularly-imprinted polymers grafted from silica capillaries have gained attention as novel affinity stationary phases in capillary electrochromatography [14,15]. In particular, MIP-based CEC can be considered a route to prepare surface polymeric thin films able to bind selectively well-defined template molecules. Due to the reduced capillary volume, MIP thin films require only very small quantities of reagents to be prepared, and the binding properties of the imprinted films can be assessed by CEC analysis with very low concentrations of the template being injected. Accordingly, this approach can be considered an effective method for investigating the binding properties of imprinted thin films [16].

**Figure 1:**
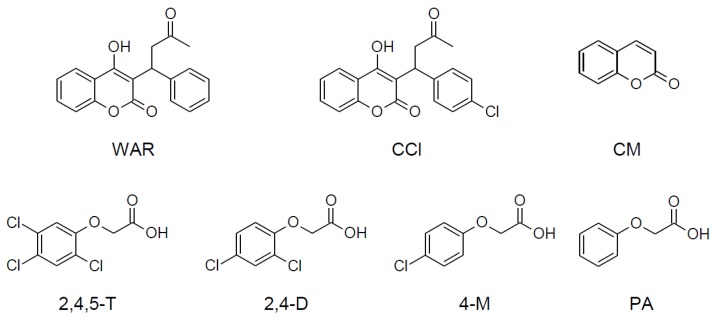
Structures of template molecules and related substances considered in this work.

This work aims to explore this possibility by investigating the potential of two different approaches for the preparation of molecularly imprinted thin layers onto CEC silica capillaries by two different surface polymerization strategies: one based on the use of 4,4′-azobis(4-cyanovaleric acid) as a surface-coupled radical initiator, the other performed with S-carboxypropyl-S’-benzyltrithiocarbonate as a RAFT agent combined with 2,2′-azobisisobutyronitrile as a free radical initiator. To verify the ability of both surface imprinting approaches to generate thin layers, two different polymerization systems, both already described in the literature [17,18], were considered: (i) a 4-vinylpyridine/ethylene dimethacrylate (4VP-EDMA) in methanol-water solution with 2,4,5-trichlorophenoxyacetic acid as a template; and (ii) methacrylic acid/ethylene dimethacrylate (MAA-EDMA) in chloroform solution with warfarin (4-hydroxy-3-(3-oxo-1-phenylbutyl)-2*H*-chromen-2-one) as a template molecule. Finally, the characterization of polymer thin layer-ligand recognition properties was explored by comparing the recognition of the templates and a series of closely-related analogues (Figure 1) by CEC analysis.

## 2. Materials and Methods

### 2.1. Chemicals and Materials

3-Aminopropyltrimethoxysilane (APTS), 4,4′-azobis(4-cyanovaleric acid) (ACVA), 2,2′-azobis(2-methylpropionitrile) (AIBN), 4-chlorophenoxy acid (4-CPA), coumachlor (CCl), coumarin (CM), 2,4-dichlorophenoxy acid (2,4-D), *N*,*N*’-diisopropylcarbodiimide (DIC), ethylendimethacrylate (EDMA), hexamethyldisilazane (HMDS), *N*-hydroxysuccinimide (NHS), methacrylic acid (MAA), phenoxycetic acid (PA), 2,4,5-trichlorophenoxy acid (2,4,5-T), 4-vinylpyridine (4-VP), warfarin (WAR), organic solvents and salts were purchased from Sigma-Aldrich-Fluka (Milan, Italy). The RAFT initiator S-carboxyethyl-S’-benzyltrithiocarbonate (CEBTTC) was synthesized according to literature [19]. All buffers and aqueous solutions were prepared with ultrapure water obtained from a Purelab Prima System from Elga (Bucks, UK) and filtered before use through a 0.22 µm cellulose acetate filter (Alltech, Milano, Italy). Toluene and acetone were dried by overnight treatment with molecular sieves previously heated to 250 °C. DMF, 4-vinylpyridine, and methacrylic acid were distilled at reduced pressure immediately before use. EDMA was purified on a basic alumina column. Stock solutions of templates and related molecules at 1.0 mg∙mL^−1^ were prepared in acetonitrile and stored in the dark at 4 °C until use. Fused silica capillaries obtained from Polymicro Technologies Optronis GmbH (Kehl, Germany) were 33 cm in length (24.5 cm to the detector) with an I.D. of 75 µm. The capillary washing reservoir CWR-10 (Supelco, Milano, Italy) was used for the capillary modification and washing procedures by applying a nitrogen positive pressure of 2 bar to allow the solution to enter the silica capillary. All the electrochromatographic separations were performed using an Agilent CE System (Agilent Technologies, Santa Clara, CA, USA) equipped with a UV-diode array detector and an Agilent ChemStation was employed for data acquisition and signal processing.

### 2.2. Silica Surface Functionalization

Bare silica capillaries were etched by flushing 1 mol∙L^−1^ sodium hydroxide for 30 min and 0.1 mol·L^−1^ hydrochloric acid for 5 min. Capillaries were then sealed and kept for two hours at 70 °C. They were subsequently flushed with water and acetone for 15 min and dried in a stream of nitrogen for 5 min. Silica was dehydrated by filling capillaries with a 10% *v*/*v* solution of DIC in anhydrous toluene and left to react overnight at room temperature. Capillaries were thereafter sequentially washed with toluene and acetone for 15 min, filled with 10% *v*/*v* APTS solution in acetone and left overnight at room temperature. They were then washed with anhydrous acetone and dried with nitrogen. After the silanization and end-capping steps were performed by filling the capillary with a 10% *v*/*v* HMDS solution in acetone and being left to react overnight at room temperature. Finally, capillaries were washed with acetone for 15 min and dried under a stream of nitrogen.

### 2.3. Silica Surface Derivatization with Azo-Initiator and RAFT Agent

Ten milligrams of ACVA or CEBTTC were converted into activated esters by reaction with 4 mg of NHS and 6.0 µL of DIC solution in 0.5 mL of distilled DMF for one hour at room temperature. The mixture was filtered through 0.22 µm polypropylene membranes to remove the precipitate. The solution was then pumped into the capillaries and left to react for 4 h at room temperature with the capillary ends capped with silicon stoppers. The capillary was finally washed with DMF and then with a 1 + 1 *v*/*v* ethanol/water solution.

### 2.4. Synthesis of MIP Thin Layers

A pre-polymerization mixture for 2,4,5-T imprinting was prepared in a 4-mL glass vial by dissolving 5.0 mg (19.6 µmol) of 2,4,5-T (template) into 2.28 mL of methanol. Subsequently, 10 µL (92.7 µmol) of 4-VP, 119 µL (631 µmol) of EDMA and 760 µL of water were added. The pre-polymerization mixture for warfarin imprinting was prepared in a 10-mL glass vial by dissolving 9.53 mg (30.9 µmol) of warfarin (template) into 4.90 mL of chloroform. Then, 8.5 µL (100 µmol) of MAA and 84 µL of EDMA (445 µmol) were added. For each template two final different polymerization mixtures were prepared: one for AICV-functionalized capillary and the other for RAFT-functionalized capillary. To the latter pre-polymerization mixture 1.5 mg of AIBN (corresponding to 1.3% of the number of vinylic groups) was added to the solution. Pre-polymerization mixtures were purged with nitrogen for 5 min and pumped into capillaries. The polymerizations were performed overnight by immersion of the sealed capillaries in a water bath at 70 °C. Afterwards, the capillaries were washed exhaustively with a solution composed by methanol-acetic acid 9 + 1 *v*/*v*. Non-imprinted capillaries were prepared with the same procedure though on the absence of template.

### 2.5. Capillary Electrochromatography

The capillary was rinsed with acetonitrile for 2 min and with the run buffer for 3 min prior to each run. The hydrodynamic injection of the sample was performed by applying 15 mbar for 2 s. The capillary cartridge was maintained at 25 °C and the absorbance was recorded at 205 nm. The separation voltage was set at +25 kV and an external pressure of 2 bar was applied to each vial to avoid bubble formation. 

For the phenoxyacid-imprinted capillary the run buffer was constituted by 5 mmol∙L^−1^ phosphate buffer, pH 2.0, containing acetonitrile in variable amounts. For the warfarin-imprinted capillary the run buffer was made by 5 mmol∙L^−1^ phosphate buffer, pH 6.4, with acetonitrile in variable amounts. The electrosmotic flow (EOF) was measured by injecting acetone or by adding it to the sample analyzed at a concentration of 1% (*v*/*v*). Injections of template and related compounds were performed after dilution to 50 mg·L^−1^ using the running buffer. Each run was repeated at least three times to assure the repeatability of the measurements. Non-imprinted capillaries were studied under the same experimental conditions as the imprinted ones.

Migration times were calculated as *t*′ = *t*_m_ − *t*_EOF_, where t_m_ is the migration time of the template and *t*_EOF_, is the migration time of the electrosmotic flow marker measured by using acetone. As *t*′ for a template molecule depends on both the electrophoretic behaviour and the interaction with the imprinted layer, its magnitude can be directly related to the strength of the molecular recognition interaction. Selectivity was calculated as α = *t*′_analogue_/*t*′_template_, where *t*′_template_ is the reduced migration time for the template and *t*′_analogue_ is the reduced migration time for a related molecule. The α-value can, in turn, be related to the difference in the change in Gibbs free energy (ΔΔG) for the binding of two different substances as calculated by using ΔΔG = −RT ln α [20].

## 3. Results and Discussion

### 3.1. Approaches to Surface Grafting

**Figure 2:**
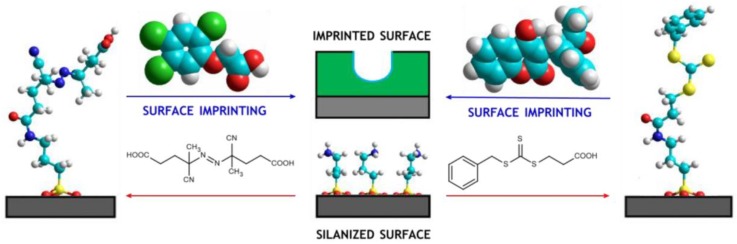
Scheme of the silica surface functionalization by ACVA (**left**) and CEBTTC (**right**) initiators.

In the present study, 2,4,5-T and warfarin were used as model templates to prepare imprinted thin layers grafted on the inner surface of 75 µm-silica capillaries (Figure 2). Both molecules have been previously described in the literature as templates of imprinted materials obtained by bulk polymerization [17,18]. In order to prevent the clogging of small-bore capillaries, the relative amount of porogenic solvent was largely increased. 

Surface-confined imprinting was fulfilled by generating a high concentration of radicals very close to the inner silica wall through the functionalization of the aminated silica surface with non-living radical initiator 4,4′-azobis(4-cyanovaleric acid), or RAFT initiator S-carboxyethyl-S’-benzyltrithiocarbonate that is able to work in living polymerization conditions. The two grafting approaches were both used with two different pre-polymerization systems in order to study the binding behaviour of imprinted thin layers obtained in markedly different experimental conditions. Preliminary evaluation of the running performance revealed that living polymerization-based imprinted capillaries for 2,4,5-T (with the initiator CEBTTC) and non-living polymerization-based imprinted capillaries for warfarin (with the initiator ACVA) were not functional. In particular, unstable run current and very limited reproducibility of the electrosmotic flow were observed. The reactivity of a mixture of monomers introduced into a narrow silica capillary and subjected to radical polimerization is notoriously very different from the corresponding bulk reactivity because of many experimental variables. As it is very difficult advancing robust hypotheses without a panel of dedicated experiments, we decided not to elaborate on this aspect further. Accordingly, the experimental work focused on the two functioning systems only.

### 3.2. Study of Polymer-Ligand Recognition Properties

Once capillaries had been prepared and their electrosmotic flows evaluated, studies were performed to establish run conditions suitable for assessing the recognition properties of the polymers for their corresponding template molecules and structurally related analogues. In affinity-CEC based on imprinted capillaries, the presence of molecular recognition properties related to the imprinting process determines the electrophoretic behaviour of the template [16,21,22]. In particular, under the same conditions, e.g., pH, ionic strength, organic modifier concentration, template-polymer molecular interactions lead to different observed relative migration times for the template or analogues on imprinted, or non-imprinted, capillaries, provided that the same separation conditions in terms of pH, ionic strength, and presence of organic solvent are carefully maintained.

#### 3.2.1. 2,4,5-T-Imprinted Capillary

The binding behaviour was studied at pH 2.0 to ensure full protonation of the 2,4,5-T, and conditions comparable to those anticipated during the polymerization, where the presence of the dissociated form can be considered negligible [17]. Under these conditions, EOF measurements showed that electrosmotic flow had an anodic direction (reversed polarity), which was attributed to the 4-vinylpyridine residues (pk_a_ ~ 6) being highly protonated, making the inner capillary surface positively charged.

**Figure 3:**
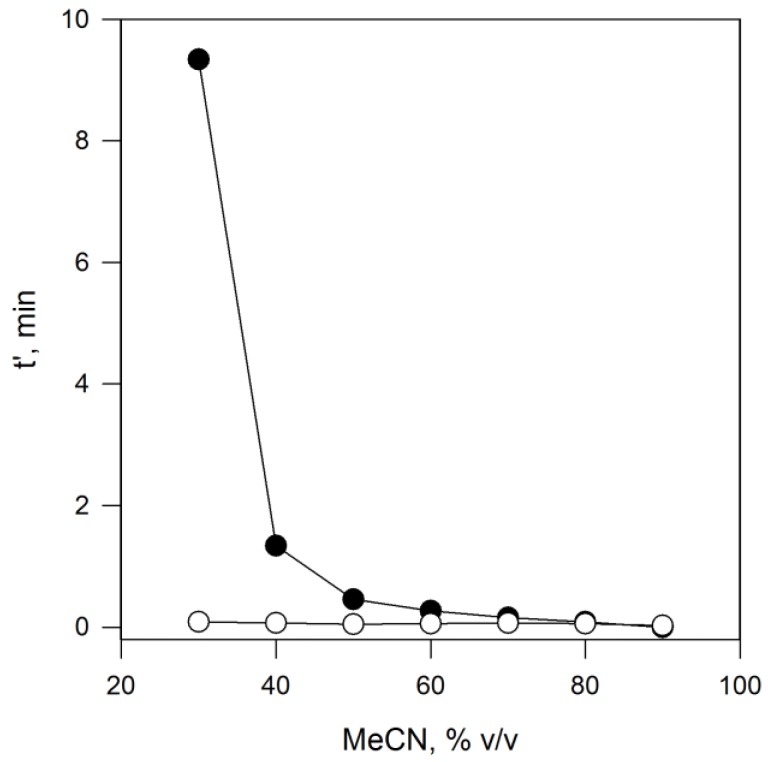
Reduced migration times (*t*′) measured in 2,4,5-T-imprinted (filled circles) and non-imprinted (empty circles) capillaries prepared with AICV initiator by using different percentages of acetonitrile added to 5 mmol∙L^−1^ phosphate buffer pH 2.0.

From the experimental data presented in Figure 3, the relative migration times measured for 2,4,5-T in the imprinted and non-imprinted capillaries were negligible, implying that the polymeric matrix has a marginal inherent capacity to recognize the template if not imprinted. A closer examination of the imprinted capillary performance revealed that even with small amounts of acetonitrile in the run buffer (<30% *v*/*v*) the relative migration for 2,4,5-T was negligible, with no response even after 30 min. Upon moving to for higher acetonitrile concentrations, the values become measurable and shorter together with an increase in the amount of acetonitrile in the run buffer. To the best of our knowledge this is the first report of this type of behaviour in a MIP-based CEC-system. We interpret this behaviour as arising from the increasing competition between analyte and organic modifier for interaction with the hydrophobic domains in the polymer film. To explain such experimental data, it is necessary to consider that acetonitrile acts a modulator of the partition mechanism between the run solution and the polymeric surface. In these conditions, the increase of acetonitrile in the run buffer interferes with the interaction between the imprinted hydrophobic pocket and the template molecule, progressively weakening its binding strength.

**Figure 4:**
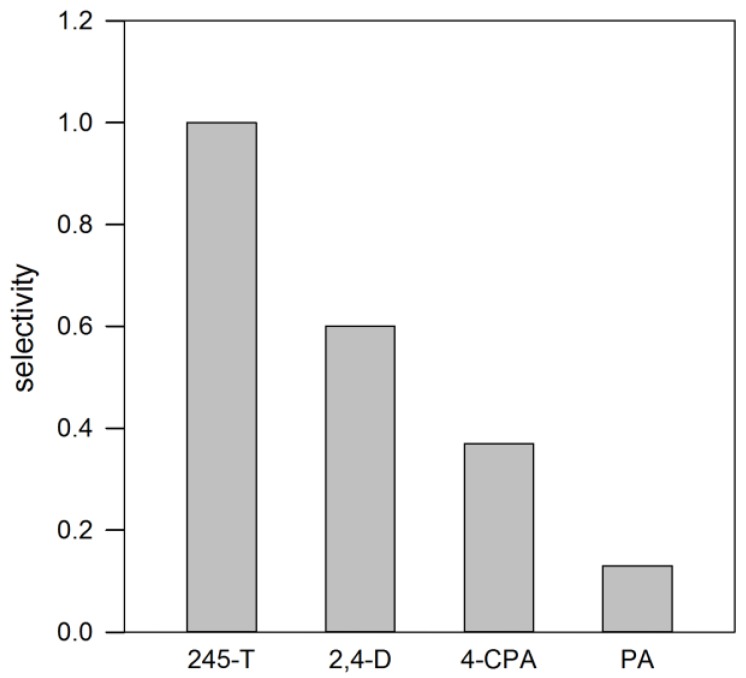
Selectivity (α) and change of Gibbs free energy (ΔΔG) of 2,4,5-T-imprinted capillary measured towards phenoxyacetic acid (PA), 4-chlorophenoxyacetic acid (4-CPA), and 2,4-dichlorophenoxyacetic acid (2,4-D) by using 50% *v*/*v* acetonitrile added to 5 mmol∙L^−1^ phosphate buffer, pH 2.0.

The selectivity of the 2,4,5-T-imprinted capillary was evaluated with several analogues differing from the template with respect to the number of chlorine substituents on the aromatic ring. The electrophoretic runs were performed by using 5 mmol∙L^−1^ phosphate buffer at pH 2.0 containing 50% *v*/*v* acetonitrile, a condition where the polymer film’s recognition of the template was observed to be still significant. The results reported in Figure 4 show that the analogues are recognized proportionally to the molecular similarity with 2,4,5-T. Thus, 2,4-D (chlorine in position 2 and 4, no chlorine in position 5) was recognized better than 4-CPA (chlorine in position 4, no chlorine in position 2 and 5), and that 4-CPA was more recognised than PA (no chlorine in position 2, 4 or 5). The plotted α-values correspond to ~1–5 kJ∙mol^−1^ in terms of change in Gibbs free energy and, thus, well compatible with small changes in the energy of interaction between ligands and imprinted binding sites due to steric and/or hydrophobic factors.

With respect to the impact of the hydrophobicity of the running buffer on recognition, a clear dependence is observed, with a significant decrease in selectivity when the buffer polarity decreases, highlighting the role of non-specific hydrophobic interactions on polymer recognition that obscure any imprinting effect, confirming the experimental results already obtained in the past by chromatography on 2,4,5-T-imprinted stationary phases [17].

#### 3.2.2. Warfarin-Imprinted Capillary

**Figure 5:**
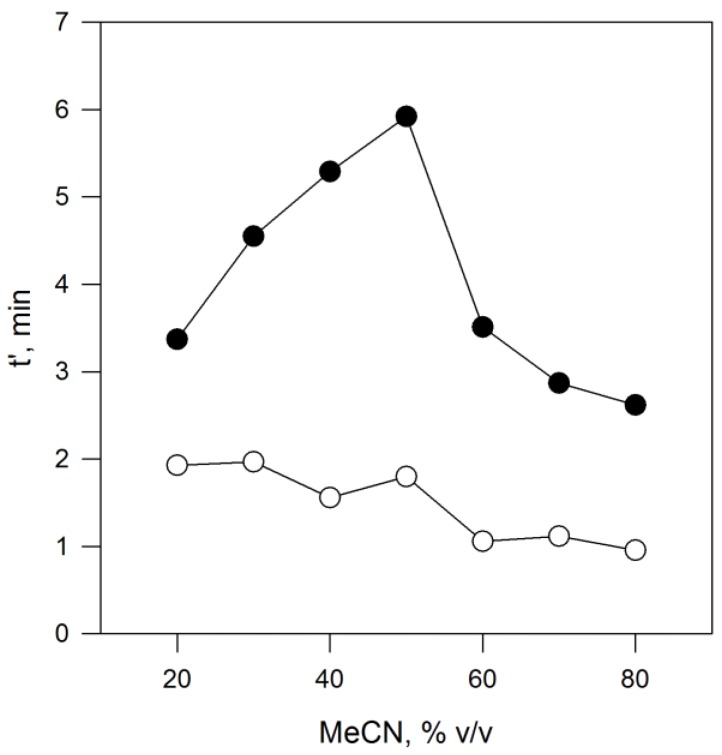
Reduced migration times (*t*′) measured in WAR-imprinted (filled circles) and non-imprinted (filled circles) capillaries prepared with RAFT initiator by using different percentages of acetonitrile added to 5 mmol∙L^−1^ phosphate buffer, pH 6.4.

The recognition behaviour was studied by using a 5 mmol∙L^−1^ phosphate buffer at pH 6.4 with various amounts of acetonitrile in order to modulate the molecular interaction between the analyte (template or analogue) and the imprinted capillary. In these conditions, the EOF measurements show that electrosmotic flow has a cathodic direction (normal polarity) because the methacrylic acid residues (pk_a_ ~ 4.7) used as functional monomers are fully deprotonated, making the inner capillary surface negatively charged. At pH 6.4 warfarin has a negative charge, and for this reason relative migration times have clear values for non-imprinted capillaries, in contrast to the case of 2,4,5-T discussed below. From the experimental data, reported in Figure 5, it is possible to see that the decrease of the running buffer polarity, by screening performance using a series of acetonitrile concentrations, causes a decrease in the relative migration time for both the capillaries, though more pronounced for the imprinted capillaries.

To make sense of such experimental data, it necessary to consider that the binding sites are a highly hydrophobic environment solvated by acetonitrile molecules, and the solvent itself competes with the template molecules for these binding sites. Thus, when acetonitrile is the main component of the running buffer, the binding sites are strongly solvated by it, and the template is less favourably bound. In contrast, when the amount of acetonitrile in the run buffer decreases at approximately 40–50% *v*/*v*, binding sites are less strongly solvated by it, increasing binding of the template. Finally, when the amount of acetonitrile in the running buffer falls under 20% *v*/*v*, the ability of the water molecules to effectively compete for formation of hydrogen bond interactions between the binding site and the template molecule prevails reducing, again, the template binding.

**Figure 6:**
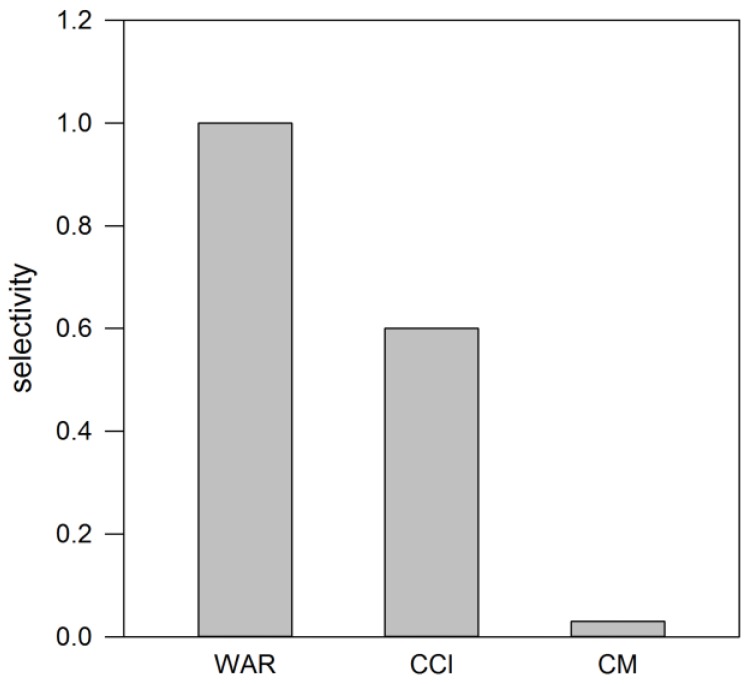
Selectivity (α) and change of Gibbs free energy (ΔΔG) of WAR-imprinted capillary measured towards coumarin (CM) and coumachlor (CCl) by using 50% *v*/*v* acetonitrile added to 5 mmol∙L^−1^ phosphate buffer, pH 6.4.

The selectivity of the warfarin-imprinted capillary was evaluated with two different analogues reported in Figure 6. The electrophoretic runs were performed under the observed optimal conditions with acetonitrile 50% *v*/*v*. The results show that coumarin was not recognized at all in either of the capillaries, as it was co-eluted with the EOF marker. The absence of recognition could be attributed to the lack of the relatively hydrophobic and sterically significant side chain present in the structure of warfarin, as confirmed by the limited but significant change in the Gibbs free energy. It is interesting that the warfarin analogue coumachlor is markedly less well recognized by the imprinted capillary, highlighting the selectivity of this polymer system, where the addition of a single substituent as a chlorine, of volume similar to a methyl substituent, has a significant impact on the polymer’s molecular recognition properties.

## 5. Conclusions

Imprinted thin layers were successfully synthesized in fused-silica capillary columns by using an azo-initiator and a RAFT agent for the templates 2,4,5-T and warfarin, respectively. The recognition properties of molecularly-imprinted capillaries were studied by affinity-CEC and compared to corresponding non-imprinted capillaries by injecting examining the migration properties of the template and structural analogues in a series different of running buffers with a range of acetonitrile concentrations. The observed recognition properties of the thin layers were comparable to those observed for polymers in prepared in the bulk format in previous studies. These results point to the potential for using affinity-CEC as a cost and material efficient means to screen the recognition properties of imprinted layers, either for use in capillary formats, or even for extrapolation to bulk polymer formats.
